# Cost-effectiveness and budget impact of heat-stable carbetocin compared to oxytocin and misoprostol for the prevention of postpartum hemorrhage (PPH) in women giving birth in India

**DOI:** 10.1186/s12913-023-09263-4

**Published:** 2023-03-17

**Authors:** John R. Cook, Kunal Saxena, Catharine Taylor, Jeffrey L. Jacobs

**Affiliations:** 1 Economic Modeling, Complete HEOR Solutions, LLC, 199 Foley Rd, Chalfont, PA 18914 USA; 2 Center for Observational and Real-World Evidence, Merck & Co., Inc, Rahway, NJ USA; 3 Global Medical & Scientific Affairs, Merck & Co., Inc, Rahway, NJ USA; 4 Office of Social Business Innovation, Merck & Co., Inc, Rahway, NJ USA

**Keywords:** Postpartum hemorrhage, Uterotonics, Cost-effectiveness, Budget impact, Economic analysis, Health economic modeling

## Abstract

**Introduction:**

Low- and middle-income countries (LMICs) are committed to achieving the Sustainable Development Goal 3.1 to reduce maternal mortality. The Ministry of Health and Family Welfare of India recommends prophylactic uterotonic administration to every woman following delivery to reduce the risk of postpartum hemorrhage (PPH), as PPH is the leading cause of maternal mortality in LMICs, including India. In 2018, the World Health Organization first recognized heat-stable carbetocin for PPH prevention. Governments are now considering its introduction into their public health systems.

**Methods:**

A decision-tree model was developed from the public healthcare system perspective to compare the value of heat-stable carbetocin versus oxytocin and misoprostol among women giving birth in public sector healthcare facilities in India. The model accounted for differences in PPH risk and costs based on mode of delivery and healthcare setting, as well as provider behavior to mitigate quality concerns of oxytocin. Model outcomes for each prophylactic uterotonic included the number of PPH events, DALYs due to PPH, deaths due to PPH, and direct medical care costs. The budget impact was estimated based on projected uterotonic uptake between 2022–2026.

**Results:**

Compared to oxytocin, heat-stable carbetocin avoided 5,468 additional PPH events, 5 deaths, and 244 DALYs per 100,000 births. Projected direct medical costs to the public healthcare system were lowered by US $171,700 (₹12.8 million; exchange rate of ₹74.65 = US$1 from 2 Feb 2022) per 100,000 births. Benefits were even greater when compared to misoprostol (7,032 fewer PPH events, 10 fewer deaths, 470 fewer DALYs, and $230,248 saved per 100,000 births). In the budget impact analysis, India’s public health system is projected to save US$11.4 million (₹849 million) over the next five years if the market share for heat-stable carbetocin grows to 19% of prophylactic uterotonics administered.

**Conclusions:**

Heat-stable carbetocin is expected to reduce the number of PPH events and deaths, avoid more DALYs, and reduce costs to the public healthcare system of India. Greater adoption of heat-stable carbetocin for the prevention of PPH could advance India’s efforts to achieve its maternal health goals and increase efficiency of its public health spending.

**Supplementary Information:**

The online version contains supplementary material available at 10.1186/s12913-023-09263-4.

## Introduction

### Universal health coverage and maternal health

In 2015, nations worldwide committed to achieve both the 2030 Agenda for Sustainable Development (SDG) and Universal Health Coverage (UHC), representing a promise of quality health services while limiting financial burden [1]. These were bold commitments for low- and middle-income countries (LMICs), where increasing burden of disease and expanding populations are rapidly growing demand for health services, while health budgets remain underfunded [2]. Perhaps there is no greater example of country commitment to SDG and UHC than in maternal health as stated in SDG 3.1 – to reduce the Maternal Mortality Ratio (MMR) to less than 70 per 100,000 births [3]. Despite these commitments, without strengthened interventions, particularly in poorer states, reaching the country’s SDG 3.1 will remain difficult to achieve [3]. This challenge is seen in India, where despite impressive reductions in India’s MMR from 370 per 100,000 live births in 2000 to 113 in 2018, equitable access to quality maternal care remains elusive for many women during their pregnancy journey [4, 5].

### Postpartum hemorrhage and prophylactic uterotonics

Although largely preventable, postpartum hemorrhage (PPH) is the leading cause of maternal mortality, particularly in LMICs [6], with uterine atony the most frequent cause of PPH [7]. Globally, PPH affects 14 million mothers annually, and almost half a million mothers died of PPH between 2003—2009 [8]. A recent global randomized clinical trial in PPH prevention found that 9% of women who gave birth vaginally experienced PPH [9], imposing significant burden in LMICs. In India, for example, PPH accounts for 26.1% of maternal deaths [8].

The use of an effective uterotonic is universally recognized as the most important medical intervention to prevent PPH. When compared with physiological management, prophylactic drugs reduce the risk of PPH by 66% [10]. Based on this evidence, the World Health Organization (WHO) recommends use of an effective uterotonic to prevent PPH during the third stage of labor for all births [11]. In 2018, a Cochrane Review was conducted to identify the most effective uterotonic agent(s) to prevent PPH with the most limited adverse events [12]. Although oxytocin is the most widely used agent globally, the network meta-analysis (U-NMA) showed that other therapies – oxytocin plus misoprostol and oxytocin/ergometrine combinations and carbetocin (stand-alone) – are associated with higher efficacy. However, the two combination regimens were associated with increased side effects, whereas carbetocin had no increase in side effects as compared with oxytocin. As for misoprostol alone, the U-NMA suggests that it is both less effective in preventing PPH compared to the above alternatives, including oxytocin, and has undesirable side-effects [12]. Despite this new network meta-analysis, oxytocin and misoprostol remain the two most widely used uterotonics to prevent PPH.

In practice, the quality of these two most widely administered uterotonics in LMICs may be poor due to manufacturing or degradation during transport and storage or exposure to climactic conditions, thereby compromising their effectiveness in preventing PPH. Because oxytocin requires constant refrigeration [13], suboptimal storage conditions in LMICs have led to widespread reports of deterioration of the quality [14–16]. The quality of misoprostol is also adversely impacted by high humidity in LMICs and poor packaging. In 2020, a systematic review across 40 LMICs further confirmed that oxytocin and misoprostol are often of poor quality with nearly 40% of each failing quality tests [17].

The updated 2018 WHO recommendations recognized alternative uterotonics when oxytocin quality was circumspect [11]. Among the alternatives, heat-stable carbetocin was included for the first time based on its efficacy, safety, and heat-stability characteristics. With a shelf-life of 48 months at 30 °C, the heat-stable formulation of carbetocin can withstand extended exposure to higher temperatures [18]. Unlike oxytocin [19] and misoprostol [12, 19], heat-stable carbetocin does not require refrigeration nor is it susceptible to humidity, and therefore seems an appropriate alternative for use, to mitigate aforementioned concerns, particularly in LMICs [13].

### Health technology assessment

Access to an effective and high-quality uterotonic to prevent PPH is a key component to achieving maternal health and UHC goals. Country prioritization of maternal health typically takes form through programs that provide free or subsidized access to maternal services, including medicines. Because LMIC governments are generally fully responsible for procurement and provision of these commodities, LMIC governments, such as India, are increasingly demanding health technology assessments (HTAs) – including cost-effectiveness and budget impact analyses – to assist programmatic and procurement decision-makers in evidence-based priority-setting of interventions [20, 21]. Health Technology Assessment in India (HTAIn) is one example of growing LMIC demand for evidence-based healthcare decision-making [22]. HTAs can also help challenge programming and purchasing behaviors that prioritize the up-front price of an intervention by showcasing the holistic efficiency and implications of current versus alternative interventions. For these reasons, HTA can be an invaluable tool to support SDG and UHC achievement.

As India and other LMICs seek to further address PPH as part of their SDG 3.1 and UHC commitments, assessing the updated WHO recommendation on uterotonics for the prevention of PPH as part of a formal HTA is, perhaps, warranted in support of prioritization-based decision-making. To this end, a health-economic model was developed to assess the cost-effectiveness and budget impact of prophylactic uterotonics. In this paper, the model is used to assess the potential role of heat-stable carbetocin as a new prophylactic uterotonic in India – at its public sector, subsidized price – compared to oxytocin and misoprostol, the two most widely used uterotonics for the prevention of PPH [23].

## Methods

### Cost-effectiveness model structure

**Fig. 1:**
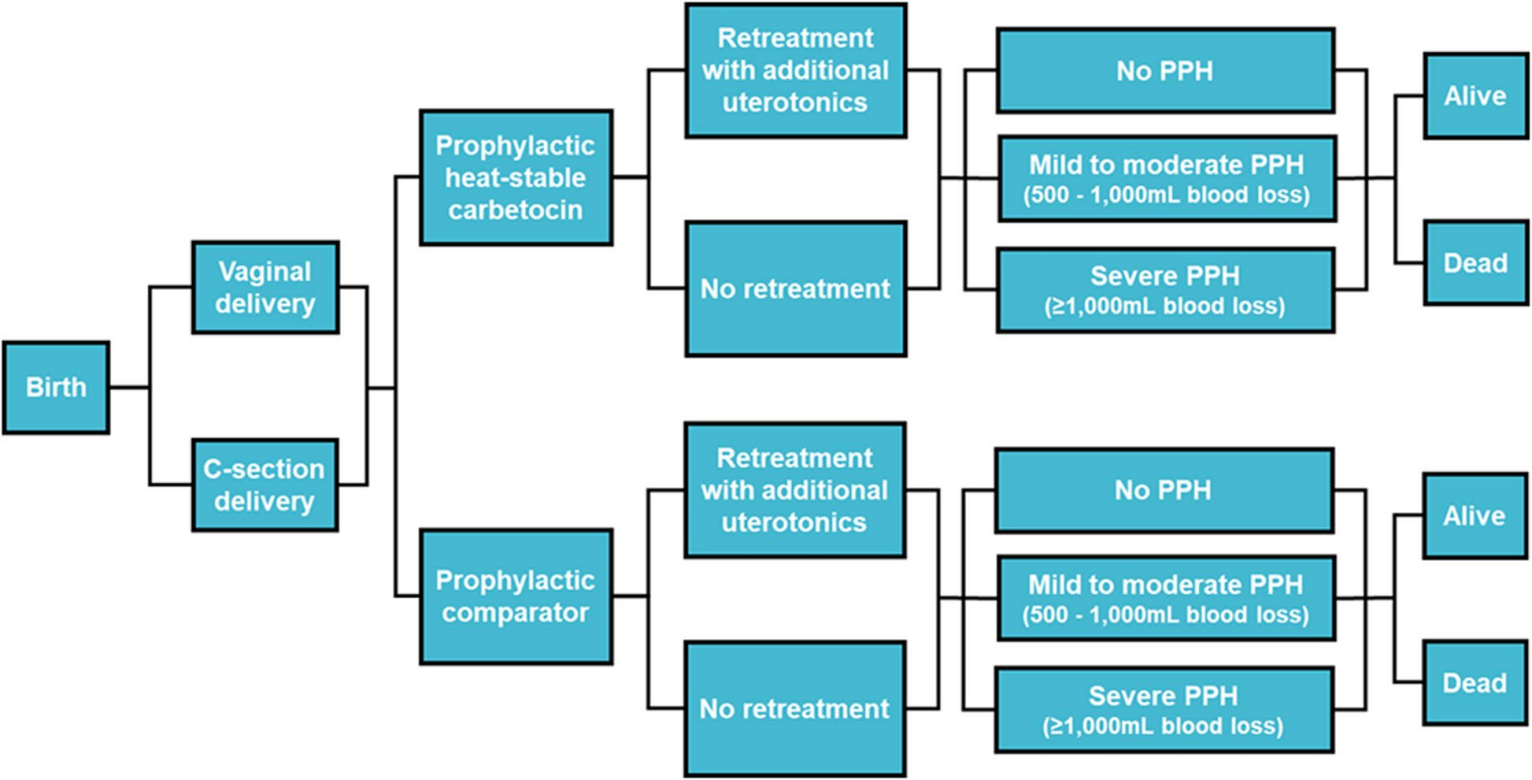
Decision tree representing possible events in women receiving prophylactic heat-stable carbetocin versus comparator (oxytocin, misoprostol) per healthcare setting

A decision tree model was developed based on the choice of uterotonic for the prevention of PPH, aligned with the WHO 2018 recommendations, to evaluate the cost-effectiveness of heat-stable carbetocin to prevent PPH compared to oxytocin and misoprostol, the two most widely used prophylactic uterotonics, from India’s public healthcare system perspective. Figure 1 shows the possible paths for women through the decision tree. Women receive a prophylactic uterotonic immediately after vaginal or caesarean section delivery (C-section), with some women requiring retreatment with additional uterotonics to treat uterine atony. Despite the use of a prophylactic uterotonic, some women may experience a PPH event that is classified as either mild/moderate PPH (500–1000 mL blood loss) or severe PPH (≥ 1000 mL blood loss). The risk of suffering either a mild/moderate or severe PPH differs by method of delivery, with C-section delivery of higher risk. Women with severe PPH are at greatest risk of death and often require transfer to a higher-level health care facility. Because the risk of PPH, death due to PPH, and costs differ by healthcare setting (primary, secondary, or tertiary facility), the decision tree is replicated for each setting in the model. Results from each tree are then weighted based on the proportion of deliveries occurring in each healthcare setting to obtain the overall results for the prophylactic uterotonic.

### Model inputs

Inputs for the model were sought from published literature, with local data utilized when available. When no referenceable source was identified, inputs were based on expert opinion from Indian clinical experts.

#### Population and delivery characteristics

We considered a cohort of 100,000 women giving birth in India across primary (PHC; includes both primary health centers and community health centers), secondary (SHC; includes District and sub-District hospitals) or tertiary (THC; includes Regional/Central-level institutions and Super specialty hospitals) public healthcare facilities, with characteristics provided in Table 1. Of these deliveries, 14.3% are performed via C-section [24], with the remaining 85.7% vaginal deliveries. The age distribution of women giving birth at a healthcare center was taken from the Indian state of Karnataka, assumed to be representative of the broader Indian population [25]. The percentage of pregnant women with anemia (hemoglobin ≤ 110 g/L) was obtained from key opinion leaders. Estimates differed by healthcare setting, with the highest (70%) and lowest (30%) percentage with anemia found in primary and tertiary healthcare centers, respectively. These estimates were consistent with an overall estimate of 50% based on data from The World Bank Group [26] and with the 2019–2021 Indian National Family Health Survey—5. The percentage of women in India with severe anemia (hemoglobin ≤ 90 g/L) was derived by healthcare setting based on an estimate that 9.4% of women with anemia experience severe anemia [27].

**Table 1:** Model inputs for delivery and women characteristics

Input Parameter		Estimate (%)	Source
Healthcare Setting	Primary	44%	Guttmacher Institute (2016) [28]
	Secondary	32%	
	Tertiary	24%	
Type of Delivery	C-section	14.3%	IIPS (2021) [24]
	Vaginal	85.7%	
Age of women (years)	15–19	2.2%	eJanMa (2015) [25]
	20–24	54.8%	
	25–29	31.6%	
	30–34	8.8%	
	35–39	2.1%	
	40–44	0.4%	
	45 and above	0.1%	
Women with anemia (Hgb ≤ 110 g/L) by healthcare center	Primary	70%	Indian clinical experts
	Secondary	50%	
	Tertiary	30%	
Women with severe anemia (Hgb ≤ 90 g/L) by healthcare center	Primary	6.6%	Nyfløt et al. (2017) [27]
	Secondary	4.7%	
	Tertiary	2.8%	

#### Efficacy of Prophylactic Uterotonics

The efficacy of prophylactic uterotonics was based on results from a 2018 Cochrane Review of uterotonic agents for preventing postpartum hemorrhage: a network meta-analysis (U-NMA) [12]. Data were taken from 135,559 women in the third stage of labor from 196 trials across 53 high-, middle- and low-income countries. Women undergoing both vaginal and caesarean births were included. Most trials were carried out in hospital settings with women undergoing vaginal births. Primary outcomes were the relative effects and rankings of each uterotonic for prevention of mild/moderate PPH and of severe PPH.

Based on a study by Nyfløt et al., pregnant women with severe anemia (hemoglobin ≤ 90 g/L) are at elevated risk of severe PPH, with an odds ratio of 4.27 (95% confidence interval: 2.79, 6.54) [27]. Because the risk of severe anemia in India differs by type of healthcare facility, an adjustment was made to the efficacy estimates from the U-NMA to account for this difference. We first estimated the risk of PPH with and without severe anemia based on the estimated percentage of women in the U-NMA with severe anemia (2.82%, or 9.4% of the 30% with anemia are severe). Next, these estimates were weighted based on the percentage of women with and without severe anemia by healthcare center in India (from Table 1). Because the risk of severe anemia is the same for the U-NMA and tertiary healthcare centers in India (2.82%), the PPH risks in a tertiary healthcare center are taken directly from the U-NMA, while the risks in a primary and secondary healthcare center are higher due to the higher percentage of women with severe anemia in these settings (see Table 2). Data from the U-NMA was also used for the proportion of women requiring re-treatment and the proportion of women requiring blood transfusions is also shown in Table 2.

**Table 2:** Risk of PPH^a^, use of additional uterotonics and blood transfusions (based on Cochrane Review U-NMA)

Intervention	Risk of PPH^a^						Additional uterotonics	Blood transfusions
	**Primary** **Healthcare Center**^**b**^		**Secondary** **Healthcare Center**		**Tertiary** **Healthcare Center**			
	**Mild / moderate PPH**	**Severe PPH**	**Mild / moderate PPH**	**Severe PPH**	**Mild / moderate PPH**	**Severe PPH**		
*Vaginal delivery*								
Heat-stable carbetocin	6.40%	2.87%	6.25%	2.73%	6.1%	2.6%	5.2%	1.2%
Oxytocin	9.60%	3.30%	9.40%	3.15%	9.2%	3.0%	11.6%	1.5%
Misoprostol	10.00%	3.96%	9.80%	3.78%	9.6%	3.6%	12.1%	1.3%
*C-section delivery*								
Heat-stable carbetocin	32.19%	12.48%	32.04%	12.04%	31.9%	11.6%	13.7%	6.6%
Oxytocin	47.13%	14.26%	47.12%	13.78%	47.1%	13.3%	30.4%	8.1%
Misoprostol	49.27%	16.85%	49.33%	16.33%	49.4%	15.8%	31.6%	7.1%

^a^Risk of PPH is adjusted for percentage with severe anemia by healthcare center type (PHC: 6.6%, SHC: 4.7%, THC: 2.8%)

^b^It is recognized that few PHC facilities in India conduct C-section deliveries (likely in only some community health centers). The model is showing the risk of PPH based on additional risk factors at PHC level, including the risk of not being transferred when indicated

#### Healthcare resource use and costs

The model captured direct costs to the healthcare system, which included drug costs for acquisition of the drug itself (initial prophylactic and additional uterotonics for PPH treatment), administration and cold-chain costs, as well as costs for healthcare providers, blood transfusions, hospitalizations, and referrals. The acquisition cost of US$0.38 (₹28.34; exchange rate of ₹74.65 = US$1 from 2 Feb 2022 [29]) for heat-stable carbetocin was based on a single-time administration through an IM/IV injection dose of 100 µg [23]. For oxytocin, while the Ministry of Health and Family Welfare (MoHFW) recommends 10 IU, the model used an average prophylactic dose administered of 17.28 IU (to account for local provider behavior that regularly seeks to mitigate quality concerns by increasing the dose) [30] at an acquisition cost of $0.24 (₹17.78) per 10 IU (Karnataka Antibiotics Pvt Ltd). For misoprostol, following MoHFW guidance, a prophylactic dose of 600 µg, priced at an acquisition cost of $0.015 (₹1.12) per 200 µg tablet, was applied. For those requiring additional uterotonics to treat PPH, the Indian clinical experts assumed 75% of women would receive oxytocin alone (40 IU) and 25% of women would receive oxytocin (40 IU) with misoprostol (800 µg) regardless of healthcare center type. All acquisition costs included 12% GST and 14% for channel partner and distribution.

Drug administration and logistics costs are also included. Administration costs for heat-stable carbetocin and oxytocin included the cost of syringe ($0.035) and 5 min of nurse/midwife time (at an average monthly salary of $380); for misoprostol, 2 min of nurse/midwife time as estimated per local experts. Cold-chain costs for logistics ($0.076) and storage ($0.049) per ampoule, and wastage (32%) were included related to oxytocin [31, 32].

Healthcare resource utilization costs associated with personnel, hospital stay, blood transfusion, referrals and follow-up care were captured for each birth by healthcare center type based on Indian clinical expert insights and inflated to 2021 costs (see Supplementary materials 1, Table S1 for additional cost input details).

#### Risk of mortality

PPH impact on mortality is also captured in the model. However, estimates for the risk of mortality due to PPH are not directly available for India. Instead, the MMR and the percentage of deaths due to PPH were used to obtain the expected number of deaths due to PPH per 100,000 women. This number was then divided by the estimated number of PPH events per 100,000 women, which is determined based on the efficacy for each prophylactic uterotonic and their respective market share from the first year in the budget impact model. For India, the MMR is 113 (2018 estimate) [4], with 26.1% of the deaths due to PPH (estimate for Southern Asia) [8]. Based on an assumed current market share of 80% oxytocin and 20% misoprostol, the resulting overall risk of mortality due to PPH is 0.16%.

The model required risk of death due to PPH stratified by severity of PPH and type of healthcare center. These were obtained through calibration (to obtain the overall risk of 0.16%) by using the odds ratios for mortality for several factors (maternal age, type of healthcare facility, actual referrals to another facility and risk of death due to referral and severity of PPH). Insights from Indian clinical experts were used to obtain the likelihood of mortality associated with severe PPH events relative to mild/moderate PPH events (estimate of 3.46). The odds ratio for mortality during referral to another hospital is estimated to be 13.35 [33]. According to local experts, it is assumed that all women experiencing a severe PPH event while in a PHC or SHC facility should be referred to a SHC or THC facility, respectively, with 45% of them actually transferred. Odds ratios for the risk of mortality were obtained from Tort et al. (2015) for healthcare setting (2.43 for PHC; 1.49 for SHC; 1.00 for THC) and maternal age (1.00 for ages less than 20; 1.48 for ages between 20 and 35; 2.16 for ages above 35) [33]. The resulting estimates for the risk of mortality due to PPH stratified by healthcare setting and severity of PPH used in the model are shown in Table 3.

**Table 3:** Risk of mortality following PPH event by severity and healthcare setting

PPH Severity	Healthcare Setting		
	PHC	SHC	THC
Mild/Moderate	0.04%	0.02%	0.01%
Severe	0.80%	0.49%	0.05%

#### Disability-adjusted life years

Disability-adjusted life years (DALYs), the preferred health outcome by WHO for presenting results of cost-effectiveness [34], were estimated for each prophylactic treatment based on the number of non-fatal and fatal PPH events. For non-fatal PPH events, DALYs were estimated based on the magnitude of the disability and its duration. The duration of disability due to mild/moderate PPH (30 days) and severe PPH (90 days) were obtained from Lubinga et al. (2015) [35], Seligman et al. (2006)[36] and Indian clinical experts. Disability weights for mild/moderate (0.166), and severe PPH (0.473 for days 0–30, 0.324 for days 31–90) were taken from the Global Burden of Disease study [37].

DALYs were also accumulated from life years lost due to premature death following a fatal PPH events. For each death, remaining life years lost was estimated using life table information for females in India [38] and the maternal age at delivery. Because DALYs due to fatal-PPH events accumulate in the future, life years lost were discounted 3% per year.

### Model output

#### Cost-effectiveness

To support assessment and decision-making related to heat-stable carbetocin as a new alternative for the prevention of PPH versus current uterotonics, the incremental cost, number of PPH events avoided, number of deaths due to PPH avoided and DALYs averted are obtained for heat-stable carbetocin versus oxytocin alone and misoprostol alone. The incremental cost-effectiveness ratio (ICER), as measured by the incremental cost per DALY averted, was calculated for heat-stable carbetocin vs each of the alternative prophylactic uterotonics. (Note: the ICER is not reported if heat-stable carbetocin decreases both the costs and DALYs against the comparator uterotonics, as there is no incremental cost to avert a DALY.) To declare an intervention to be cost-effective, we used a willingness-to-pay threshold equivalent to the 2020 per capita GDP in India of $1,900.75 (₹141,891), as per India’s HTAIn policy.

Robustness of the results to uncertainty in model parameters were evaluated in both one-way sensitivity analyses (OWSA, to isolate the impact of one parameter using extreme values) and probabilistic sensitivity analyses (PSA, to assess the joint impact of uncertainty in multiple parameters across the range of likely values). (See Supplementary materials 1, Table S2 for input values and distributions). Results for the PSA were based on 5,000 random sets of the parameters drawn from their statistical distribution.

Finally, scenario analyses were performed to determine the impact alternative model settings and choice of input sources have on the cost-effectiveness of heat-stable carbetocin vs oxytocin and misoprostol. The scenarios were selected based on maternal health and supply chain stakeholder interests. Four scenarios associated with cold-chain costs were evaluated, including where cost-chain costs were excluded based on the assumption that cold-chain will be required for other medicines regardless if heat-stable carbetocin is used prophylactically or not [31, 32]. Two separate scenarios, where a lower oxytocin dose (to the recommended level of 10 IU) and lower misoprostol dose (400 µg), were considered, aligning with WHO recommendations; in both scenarios, no reduction in efficacy was assumed. A scenario where the base-case acquisition costs for oxytocin and misoprostol were both reduced by 25% was analyzed to consider price sensitivity differences. A scenario reflecting the impact of using efficacy estimates for oxytocin and heat-stable carbetocin using data from CHAMPION was assessed, the largest clinical trial in PPH prevention [39]. Finally, scenarios were evaluated with alternative sources for the percentage of women with anemia [24, 26], age distribution of women giving birth [36] and the duration of hospitalization (Indian clinical experts) to support further country contextualization.

### Budget impact analysis

A budget impact analysis was also conducted by projecting the annual cost to the healthcare system for births at public institutions based on the current estimated market share of uterotonics (“current environment”) and based on a potential shift in market share with uptake of heat-stable carbetocin (“new environment”) over the next 5 years per discussion with local experts in India (see Table 4). The budget depends on the total number of births from 2022 through 2026, which was estimated using a crude rate of 18.083 births per 1,000 persons in a population of 1,352,617,328 in 2018 with an annual growth rate of 1.04% [26]. Of these births, 88.6% are institutional births, of which 61.9% occur in a public facility [24]. The distribution of births by healthcare facility type and type of delivery, along with clinical and healthcare resource use inputs are the same as used in the cost-effectiveness analysis.

**Table 4:** Market share of prophylactic uterotonics under current and new environment (2022–2026)

Intervention	2022	2023	2024	2025	2026
Current Environment (without Heat-Stable Carbetocin)					
Oxytocin	80%	80%	80%	80%	80%
Misoprostol	20%	20%	20%	20%	20%
New Environment (with Heat-Stable Carbetocin)					
Heat-stable carbetocin	0.2%	2%	6%	13%	19%
Oxytocin	79.8%	78%	75%	69%	63%
Misoprostol	20%	20%	19%	18%	18%

## Results

### Cost-effectiveness analysis

In the comparison versus oxytocin, administering heat-stable carbetocin for PPH prevention resulted in fewer PPH events (28.0% reduction overall), deaths and DALYs. In the comparison versus misoprostol, administering heat-stable carbetocin for PPH prevention resulted in an even greater reduction in PPH events (33.3% reduction overall), deaths and DALYs. (See Table 5 for results for the three prophylactic uterotonics).

**Table 5:** Total PPH events, deaths, DALYs and Costs per 100,000 births

Intervention	Total PPH events	Severe PPH events	Deaths	DALYs	Total cost (in $)	Total cost (in ₹)
Heat-stable Carbetocin	14,070	4,100	25	1,170	13,990,033	1,044,355,971
Oxytocin	19,538	4,711	30	1,414	14,161,733	1,057,173,404
Misoprostol	21,102	5,618	35	1,640	14,220,281	1,061,543,962

Total costs to the public healthcare system are lowest when heat-stable carbetocin is selected as the prophylactic uterotonic administered (see Table 5). These cost benefits can largely be attributed to the greater efficacy of heat-stable carbetocin in preventing more PPH events. A breakdown of costs by category are provided in Supplementary materials 2 (see Table S3) and reveals savings with heat-stable carbetocin across all cost categories except follow-up costs (which are assumed to be the same) compared to oxytocin and across hospital-related costs (hospital stay, personnel, blood transfusions and referrals) compared to misoprostol.

From a cost-effectiveness standpoint – combining health outcomes and healthcare costs – heat-stable carbetocin out-performs both oxytocin and misoprostol from the public healthcare system perspective in India, as it lowers costs and DALYs are averted. This result is observed across each of the three healthcare facility types (see Supplementary materials 2, Table S4). In the comparison versus oxytocin, health outcomes for heat-stable carbetocin are improved the most for births in the PHC (e.g., 0.003 DALYs averted per woman), while the cost reduction is the greatest in the THC ($2.42 decrease per birth).

#### Sensitivity analyses

The OWSA were conducted comparing oxytocin vs each of the prophylactic uterotonics separately for the incremental DALYs and incremental costs. The resulting tornado diagrams show the 10 most influential parameters for both measures (see Supplementary materials 3, Figures S1 and S2). Across all comparisons, the parameters with the greatest impact on DALYs averted are predominantly related to mortality, referrals, and the proportion of C-section deliveries, whereas those with the greatest impact on the incremental cost are related to the duration of hospital stay. Regardless of the parameter varied, costs are always lower and DALYs are averted with heat-stable carbetocin compared to oxytocin and misoprostol.

**Fig. 2:**
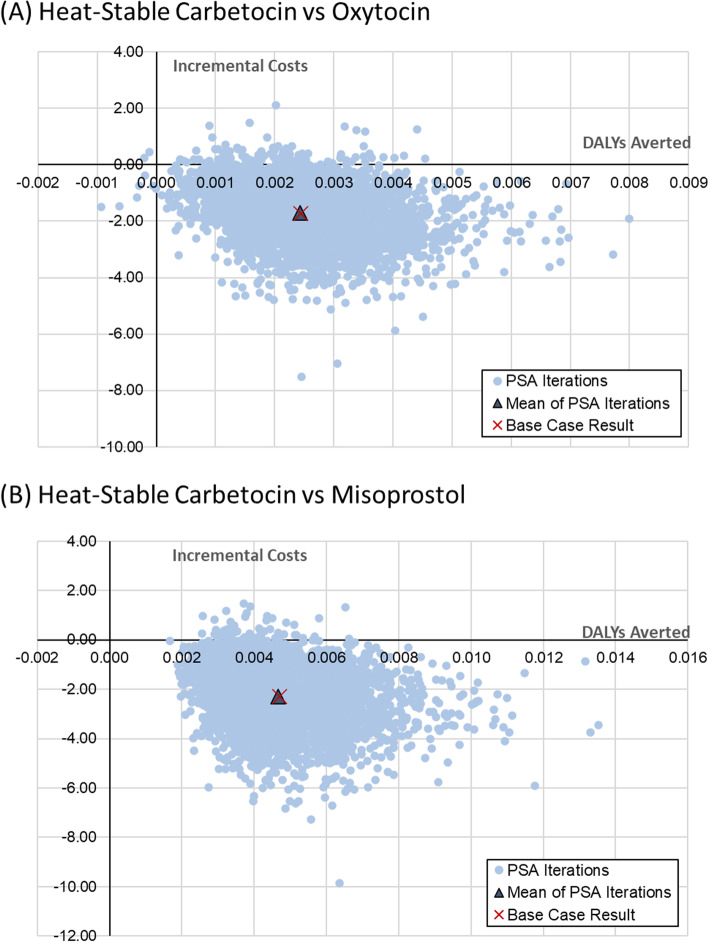
Probabilistic sensitivity analysis results for incremental costs and DALYs averted. **A** Heat-stable carbetocin versus oxytocin and (**B**) Heat-stable carbetocin versus misoprostol

Results of the PSA are displayed in Fig. 2 with the PSA replicates of the incremental costs and DALYs avoided for heat-stable carbetocin versus each prophylactic uterotonic. Heat-stable carbetocin out-performs both oxytocin (Fig. 2A) and misoprostol (Fig. 2B); nearly all (98.0% and 98.9%, respectively) of the PSA replicates indicate heat-stable carbetocin lowers costs and averts more DALYs than either alternative prophylactic uterotonic.

#### Scenario analysis

Across the scenario analyses, heat-stable carbetocin continued to out-perform both oxytocin and misoprostol (see Supplementary materials 3, Table S5). Heat-stable carbetocin, as a prophylactic alternative, continued to produce the lowest costs compared to oxytocin and misoprostol, despite scenario changes:

- excluding cold-chain costs for oxytocin lowered the savings versus oxytocin from $1.72 to $1.50 per birth and versus misoprostol from $2.30 to $2.29 per birth.
- use of an alternative source for cost-chain costs resulted in savings per woman ranging from $1.67 to $2.49 per birth and $2.30 to $2.36 per birth compared to oxytocin and misoprostol, respectively, depending on the source of costs.
- setting the average dose for prophylactic oxytocin to 10 IU (recommended dose by the MoHFW of India) lowered the savings ($1.54 per birth).
- reducing oxytocin and misoprostol pricing by 25% lowered the savings to $1.57 and $2.27 per birth compared to oxytocin and misoprostol, respectively.
- replacing the source for efficacy with the CHAMPION trial resulted in savings of $0.82 per birth compared to oxytocin.

### Budget impact analysis

From 2022 through 2026, 71.4 million cumulative births were projected in public healthcare facilities in India (see Table 6) [24]. During this period, costs to the public healthcare system are projected to decrease by $11.4 million (₹849.2 million), based on incremental growth in heat-stable carbetocin integration – reaching 19% of prophylactic uterotonic use by 2026 – compared to projected costs based on current prophylactic oxytocin and misoprostol utilization patterns (without inclusion of heat-stable carbetocin). These budget savings are driven by approximately 322,000 fewer PPH events projected to occur.

**Table 6:** Projected annual budget and clinical impact of new versus current market share for uterotonics

	**2022**	**2023**	**2024**	**2025**	**2026**
Total Births	13,979,677	14,124,691	14,271,210	14,419,249	14,568,823
***Total Costs ($)***					
Current environment	1,975,973,855	1,996,471,093	2,017,180,954	2,038,105,644	2,059,247,390
New environment	1,975,920,321	1,995,930,198	2,015,491,503	2,034,415,603	2,053,845,363
**Budget impact**	-53,534	-540,895	-1,689,452	-3,690,041	-5,402,026
***Total Costs*** (₹)					
Current environment	147,506,448,280	149,036,567,126	150,582,558,253	152,144,586,306	153,722,817,641
New environment	147,502,451,954	148,996,189,314	150,456,440,696	151,869,124,773	153,319,556,370
**Budget impact**	**-3,996,326**	**-40,377,812**	**-126,117,556**	**-275,461,533**	**-403,261,272**
***Total PPH Events***					
Current environment	2,633,715	2,661,035	2,688,639	2,716,529	2,744,708
New environment	2,632,219	2,645,917	2,640,648	2,611,835	2,592,148
**Clinical impact**	**-1,496**	**-15,118**	**-47,991**	**-104,694**	**-152,560**

## Discussion

Our analysis focused on comparing a novel uterotonic – heat-stable carbetocin – to the most widely used uterotonics – oxytocin (standard of care) and misoprostol – for the prevention of PPH. We sought to assess the impact heat-stable carbetocin uptake may have on the Indian public healthcare system. Our findings show that the introduction of prophylactic heat-stable carbetocin is a cost-effective intervention driven by fewer total PPH events. These fewer PPH events – 28% to 33% fewer compared to prophylactic oxytocin and misoprostol, respectively – lead to improved overall health outcomes and, subsequently, cost savings to the Indian health system. Our findings indicate the public health system is projected to save $11.4 million (₹849 million; exchange rate of ₹74.65 = US$1 from 2 Feb 2022 [29]) over the next 5 years (2022 – 2026) if heat-stable carbetocin uptake reaches 19% of all births by 2026. Finally, our analysis suggests that if uptake of heat-stable carbetocin is more rapid, reaching, for example, 50% of all births by year 5, savings are projected to rise to $29.9 million (₹2.23 billion [36]).

Our results are consistent with recent analyses that signaled the ability of heat-stable carbetocin to prevent the greatest number of PPH events compared to oxytocin and misoprostol. Based on a model by Matthijsse et al. (2022) for the UK, carbetocin was associated with 3.42 fewer PPH events and 0.39 fewer severe PPH events per 100 women compared to oxytocin [40]. While overall rates for PPH were lower in the UK compared to India, both their model and ours suggest that roughly one out of every 9 avoided PPH events was severe. As with our model, they found carbetocin resulted in total cost savings. A similar result was found for Canada by Barrett, et al. (2022) where cost savings of carbetocin versus oxytocin were again primarily attributed to avoiding consequences of PPH [41]. In another cost-effectiveness evaluation for the UK, Pickering, et al. (2018) found carbetocin to lower the risk of total and severe PPH events, which in turn resulted in a lower total cost compared to misoprostol [42].

LMICs have committed to achieving SDG 3.1 by 2030. Although progress is being made, many countries are unlikely to meet this goal at their current pace [43]. PPH remains the leading direct cause of maternal mortality and is largely preventable. Optimizing PPH prevention efforts should be paramount and would accelerate progress toward achieving SDG 3.1. WHO’s 2018 Recommendations on Uterotonics for the Prevention of PPH provide a new alternative, heat-stable carbetocin, for countries to consider in helping to accelerate progress. Based on our evaluation, adoption of heat-stable carbetocin in India is expected to reduce PPH events, leading to a reduction in health care resource utilization and material cost-savings to India’s public healthcare system – key supportive tenets of country achievement of SDG 3.1 and Universal Health Coverage objectives.

Health care policy makers are increasingly looking to health technology assessments to support informed programmatic and procurement decisions, including the ones related to new innovations such as heat-stable carbetocin [22]. Applying WHO’s 2018 Recommendations, and considering India’s public health system, the results of our assessments provide compelling rationale for country adoption of heat-stable carbetocin as a new alternative to help optimize PPH prevention efforts.

Reducing PPH events has, as a consequence, additional clinical, institutional and societal benefits. The most straight-forward are a lower number of PPH-related near-misses, post-partum complications and deaths, and a reduction in DALYs. Fewer PPH events also has the further benefit of alleviating over-burdened public health facilities, primarily by reducing the number of affected women requiring additional hospital stay, the duration of that hospital stay and the related healthcare personnel time and cost to manage their care.

The considerable savings to the Indian public health system suggested by the budget impact analysis following the uptake of heat-stable carbetocin for PPH prophylaxis would provide the opportunity to shift limited resources to complementary or other areas of health.

Use of heat-stable carbetocin also brings advantages with respect to country implementation. As refrigeration is not required and humidity has no negative consequence, it mitigates against potential weakness in cold-chain integrity – from manufacturer to in-country transport and storage or consistency in facility access to electricity – and climactic conditions, and thus can be conveniently used in any setting while maintaining its quality. Consequently, consistency in quality can lead to better real-world outcomes and, importantly, enhanced trust between provider and patient that she is getting good quality medicine.

While the heat-stability of carbetocin may be perceived as a key advantage in more remote, primary healthcare facilities where cold-chain and electricity challenges are most prevalent, this study demonstrated benefits are achieved across all levels of the public healthcare system (PHC, SHC, THC), even when conservatively assuming quality is equalized across prophylactic uterotonics. While reductions in mortality are greater in the PHC and cost savings are higher in the THC, heat-stable carbetocin lowered DALYs and costs over both oxytocin and misoprostol regardless of the setting.

Importantly, the cost-effectiveness model also shows that overall costs savings held true despite a higher acquisition cost of heat-stable carbetocin compared to oxytocin or misoprostol, and the up-front cost of any prophylactic uterotonic – heat-stable carbetocin, oxytocin or misoprostol – was not a key driver of the overall cost to address PPH. Our analysis highlights that programmatic and procurement decisions based solely on the up-front uterotonic acquisition cost would lead to a suboptimal budget decision and would be misaligned with the goal to achieve both SDG 3.1 and UHC agendas.

It should be noted that while the assessment presented in this paper was applied to India, the model can easily be adapted using country-specific inputs to assist decision makers in any LMIC.

## Limitations

While providing insights into alternative programmatic and procurement decisions, our heath economic evaluation is not without limitations. Due to the lack of local sources for several aspects of uterotonic use and the influence of factors such as anemia on rates of PPH and death rates from PPH, some data inputs and assumptions may lack peer-reviewed validation. In these cases, we relied heavily on insights from Indian clinical experts. The impact of these data limitations is difficult to quantify, but sensitivity analyses suggest overall results (cost saving and improved health outcomes) are robust to uncertainty in model parameters. These data limitations also reinforce the need to improve data collection around maternal health care, and specifically around PPH as the leading direct cause of death.

The greater efficacy of heat-stable carbetocin to prevent PPH events compared to oxytocin and misoprostol was supported by the 2018 Cochrane Review U-NMA [12]. While the methodology is designed to minimize the risk, results are subject to potential bias due to differences in the included studies. When we utilized the alternative CHAMPION trial for the efficacy of heat-stable carbetocin and oxytocin, [39] the benefit of heat-stable carbetocin was reduced, but more PPH events were still avoided and total costs remained lower with heat-stable carbetocin. Regardless of the study used, the effectiveness of uterotonics on PPH is likely to differ in practice due to manufacturing, transport, storage, or climactic issues that can impact quality. For example, poor-quality misoprostol was not factored into the model despite documented evidence of consistently poor quality. For oxytocin, while the prophylactic recommended dosage was increased to compensate for poor-quality based on behavioral practice (and thus the costs increased), we did not decrease its effectiveness. Thus, the model is likely underestimating the risk of PPH and associated costs compared with the real-world prophylactic administration of oxytocin and misoprostol. Further, studies have shown that cold-chain is not always maintained in LMICs, and thus the true costs of cold-chain are unknown and may be under-estimated.

While we indicate maternal age is an important factor in the risk of death from PPH based on Tort et al. (2015), with risk increasing by age group (< 20, 20 to 35, > 35), results are not provided separately by age. We chose not to investigate this given the high percentage of women in the 20 to 35 age group (over 95%) and the lack of data on other factors that might differ by age (i.e., rate of c-sections). If age-based decisions regarding use of prophylaxis is to be investigated, further efforts would be needed to collect these inputs.

Finally, the costs of certain PPH management interventions (e.g., laboratory tests and invasive procedures) were excluded from the model to allow for the greatest applicability across healthcare settings, thus likely under-estimating the total medical costs to manage PPH. And, as the model only focused on direct PPH-related medical care costs to the healthcare system, additional start-up costs to the system, such as healthcare provider training on new alternatives, or out-of-pocket costs to the individual/family, were not included.

## Conclusions

The cost-effectiveness and budget impact models can be used by decision makers in LMICs to assess the utility of heat-stable carbetocin as a first line alternative to prevent PPH. Despite the limitations, this model projects a favorable impact – health outcomes and economic savings – of introducing heat-stable carbetocin in India’s public sector. Across all levels of the healthcare system, heat-stable carbetocin was projected to reduce the number of PPH events at a lower cost to the public healthcare system. While our findings are consistent with recent studies [40–42], to our knowledge this is the first time this has been shown in a LMIC setting using public health sector pricing for all uterotonics, including heat-stable carbetocin. Consequently, these results, based on Health Technology Assessment methodology, suggest heat-stable carbetocin could be a valuable new prophylactic uterotonic in India’s efforts to prevent PPH and advance its efforts toward achievement of its SDG 3.1 and Universal Health Coverage goals.

## Supplementary Information

**Additional file 1:** **Table S1.** Healthcare resource utilization cost inputs by healthcare center type. **Table S2.** Key* model input values for deterministic and probabilistic sensitivity analyses. **Table S3.** Breakdown of total costs per 100,000 women. **Table S4.** Incremental outcomes per woman for heat-stable carbetocin versus alternative prophylactic uterotonics by healthcare center type. **Table S5.** Scenario Analysis: Costs and health outcomes per woman for all births. **Fig S1.** Tornado diagrams for heat-stable carbetocin vs oxytocin. **Fig S2.** Tornado diagrams for heat-stable carbetocin vs misoprostol.

## Data Availability

Data can be acquired from the corresponding author on reasonable request.

## Abbreviations

DALYs: Disability-adjusted life years
HTA: Health technology assessment
HTAIn: Health Technology Assessment in India
ICER: Incremental cost-effectiveness ratio
LMICs: Low- and middle-income countries
MMR: Maternal mortality ratio
MoHFW: Ministry of Health and Family Welfare
OWSA: One-way sensitivity analyses
PHC: Primary health center
PPH: Postpartum hemorrhage
PSA: Probabilistic sensitivity analyses
SDG: Sustainable Development Goal
SHC: Secondary health center
THC: Tertiary health center
UHC: Universal health coverage
U-NMA: Uterotonic network meta-analysis
WHO: World Health Organization
