# Supplementary material for: Cost-effectiveness and budget impact of heat-stable carbetocin compared to oxytocin and misoprostol for the prevention of postpartum hemorrhage (PPH) in women giving birth in India
Source: BMC Health Serv Res. 2023 Mar 17;23:267. doi: 10.1186/s12913-023-09263-4 (PMC10024421; doi:10.1186/s12913-023-09263-4)
Supplement: Supplementary file 1 — Additional file 1: Table S1. Healthcare resource utilization cost inputs by healthcare center type. Table S2. Key* model input values for deterministic and probabilistic sensitivity analyses. Table S3. Breakdown of total costs per 100,000 women. Table S4. Incremental outcomes per woman for heat-stable carbetocin versus alternative prophylactic uterotonics by healthcare center type. Table S5. Scenario Analysis: Costs and health outcomes per woman for all births. Fig S1. Tornado diagrams for heat-stable carbetocin vs oxytocin. Fig S2. Tornado diagrams for heat-stable carbetocin vs misoprostol. [file 12913_2023_9263_MOESM1_ESM.docx]

**Cost-effectiveness and budget impact of heat-stable carbetocin compared to oxytocin and misoprostol for the prevention of postpartum hemorrhage (PPH) in women giving birth in India**

**Cook, et. al.**

# Supplementary materials

1. Additional model inputs for costs and sensitivity analyses

Table S1. Healthcare resource utilization cost inputs by healthcare center type

| Cost Component | Primary HC | Secondary HC | Tertiary HC |
| --- | --- | --- | --- |
| **Personnel for PPH Management** |  |  |  |
| Time per Birth (minutes) | | | |
| Physician/Doctor/Medical Officer |  |  |  |
| No PPH | 17.5 | 17.5 | 17.5 |
| Mild/Moderate PPH | 45 | 45 | 45 |
| Severe PPH | 150 | 150 | 150 |
| Nurse |  |  |  |
| No PPH | 30 | 30 | 30 |
| Mild/Moderate PPH | 75 | 75 | 75 |
| Severe PPH | 150 | 150 | 150 |
| Auxiliary Nurse Midwife |  |  |  |
| No PPH | 30 | 0 | 0 |
| Mild/Moderate PPH | 75 | 0 | 0 |
| Severe PPH | 150 | 0 | 0 |
| Salaries for Personnel ($ per month) | | | |
| Physician/Doctor/Medical Officer | 1,073 | 1,692 | 2,022 |
| Nurse / Midwives | 380 | 380 | 380 |
| Auxiliary Nurse Midwife | 281 | 281 | 281 |
|  |  |  |  |
| **Hospital Stay** |  |  |  |
| Average Length of Stay (days) |  |  |  |
| No PPH | 2 | 2 | 2 |
| Mild/Moderate PPH | 2 | 2 | 2 |
| Severe PPH | 4 | 4 | 4 |
| Hospital Cost ($ per day) | 24.76 | 61.91 | 99.06 |
|  |  |  |  |
| **Blood Transfusions** |  |  |  |
| Average Number of Units |  |  |  |
| No PPH | 0 | 0 | 1.5 |
| Mild/Moderate PPH | 2.5 | 2.5 | 2.5 |
| Severe PPH | 4 | 4 | 13 |
| Cost ($ per unit of blood) | 16.51 | 16.51 | 24.76 |
|  |  |  |  |
| **Transfer between Healthcare Centers** |  |  |  |
| % with PPH Requiring Transfer | 45% | 45% | -- |
| Cost ($ per transfer) | 24.76 | 24.76 | -- |
|  |  |  |  |
| **Follow-up** |  |  |  |
| Cost without Secondary Infection ($) | 16.51 | 16.51 | 16.51 |
| Cost with Secondary Infection ($) | 165.10 | 165.10 | 165.10 |
| Women with Secondary Infections (%) | 1.0% | 2.5% | 2.5% |

Table S2. Key* model input values for deterministic and probabilistic sensitivity analyses

| **Parameter** | **Base Case** | **One-Way Sensitivity Analysis** | | **PSA Distribution** |
| --- | --- | --- | --- | --- |
|  |  | **Lower Bound** | **Upper Bound** |  |
| **Delivery** |  |  |  |  |
| Proportion of C-section births | 0.143 | 0.092 | 0.203 | Beta (21.38,127.54) |
| **Mortality** |  |  |  |  |
| Due to mild/moderate PPH event | 0.00010 | 0.00007 | 0.00015 | Beta (25.00, 24121.76) |
| Due to severe PPH event | 0.00036 | 0.00023 | 0.00051 | Beta (24.99, 70150.36) |
| Odds Ratio: age 20-35 years (vs < 20 years) | 1.48 | 0.93 | 2.44 | Gamma (15.13, 0.10) |
| Odds Ratio: age > 35 years (vs < 20 years)** | 2.16 | 1.26 | 3.72 | Gamma (12.14, 0.18) |
| Odds Ratio: PHC (vs THC) | 2.43 | 1.29 | 4.56 | Gamma (8.70, 0.28) |
| Odds Ratio: SHC (vs THC) | 1.49 | 0.91 | 2.44 | Gamma (14.94, 0.10) |
| Odds Ratio: due to referral | 13.35 | 8.64 | 19.07 | Gamma (25.00, 0.53) |
| Proportion of referrals that get transferred | 0.45 | 0.28 | 0.63 | Beta (13.30, 16.26) |
| **Disability Weights** |  |  |  |  |
| Duration of disability: mild/moderate PPH (days) | 30 | 19.41 | 42.85 | Gamma (25.00, 1.20) |
| Duration of disability: severe PPH (days)** | 90 | 58.24 | 128.56 | Gamma (25.00, 3.60) |
| Weights: mild/moderate PPH | 0.166 | 0.11 | 0.24 | Beta (20.68, 103.92) |
| Weights: severe PPH (days 1-30)** | 0.473 | 0.29 | 0.66 | Beta (12.70, 14.15) |
| Weights: severe PPH (days 31-90) | 0.324 | 0.20 | 0.46 | Beta (16.58, 34.58) |
|  |  |  |  |  |
|  |  |  |  |  |
| **Duration of Hospital Stay (days)** |  |  |  |  |
| PHC: vaginal delivery with no PPH | 2 | 1.29 | 2.86 | Gamma (25.00, 0.08) |
| PHC: vaginal delivery with mild/moderate PPH | 2 | 1.29 | 2.86 | Gamma (25.00, 0.08) |
| PHC: vaginal delivery with severe PPH** | 4 | 2.59 | 5.71 | Gamma (25.00, 0.16) |
| PHC: C-section delivery with no PPH | 2 | 1.22 | 2.78 | Normal (2.00, 0.40) |
| PHC: C-section delivery with mild/moderate PPH | 2 | 1.22 | 2.78 | Normal (2.00, 0.40) |
| PHC: C-section delivery with severe PPH** | 4 | 2.43 | 5.57 | Normal (4.00, 0.80) |
| SHC: vaginal delivery with no PPH | 2 | 1.29 | 2.86 | Gamma (25.00, 0.08) |
| SHC: vaginal delivery with mild/moderate PPH | 2 | 1.29 | 2.86 | Gamma (25.00, 0.08) |
| SHC: vaginal delivery with severe PPH | 4 | 2.59 | 5.71 | Gamma (25.00, 0.16) |
| SHC: C-section delivery with no PPH | 2 | 1.22 | 2.78 | Normal (2.00, 0.40) |
| SHC: C-section delivery with mild/moderate PPH | 2 | 1.22 | 2.78 | Normal (2.00, 0.40) |
| SHC: C-section delivery with severe PPH** | 4 | 2.43 | 5.57 | Normal (4.00, 0.80) |
| **Cost per Day in Hospital (in $s)** |  |  |  |  |
| PHC** | 24.76 | 15.08 | 30.17 | Gamma (42.45, 0.58) |
| SHC | 61.91 | 22.63 | 90.50 | Gamma (13.10, 4.73) |
| THC** | 99.06 | 30.17 | 150.84 | Gamma (10.61, 9.33) |

* Parameters identified among the 10 most influential during OWSA for incremental QALYs or incremental Costs for heat-stable carbetocin versus either oxytocin or misoprostol

** Parameters not among the 10 most influential, but included for completeness of parameter set

1. Additional results for base case analysis

Table S3: Breakdown of total costs per 100,000 women

|  | Heat-stable carbetocin | Oxytocin | Misoprostol |
| --- | --- | --- | --- |
| *Total Costs* ($) | | | |
| Drug costs | 43,932 | 54,976 | 18,909 |
| Administration costs | 20,513 | 22,147 | 9,342 |
| Healthcare personnel costs | 551,689 | 595,989 | 622,135 |
| Hospital stay costs | 11,334,686 | 11,400,291 | 11,497,731 |
| Blood transfusion costs | 47,887 | 70,118 | 66,115 |
| Referral costs | 35,292 | 40,537 | 48,315 |
| Follow-up costs | 1,954,746 | 1,954,746 | 1,954,746 |
| Cold-chain logistics | 485 | 8,632 | 1,124 |
| Cold-chain storage | 314 | 5,586 | ,728 |
| Cold-chain wastage | 489 | 8,711 | 1,135 |
| **Total costs** | **13,990,033** | **14,161,733** | **14,220,281** |
| **Cost per delivery** | **140** | **142** | **142** |
| ***Total Costs*** (₹) | | | |
| Drug costs | 3,279,491 | 4,103,973 | 1,411,545 |
| Administration costs | 1,531,328 | 1,653,254 | 697,412 |
| Healthcare personnel costs | 41,183,618 | 44,490,572 | 46,442,380 |
| Hospital stay costs | 846,134,311 | 851,031,686 | 858,305,600 |
| Blood transfusion costs | 3,574,787 | 5,234,328 | 4,935,515 |
| Referral costs | 2,634,534 | 3,026,113 | 3,606,711 |
| Follow-up costs | 145,921,819 | 145,921,819 | 145,921,819 |
| Cold-chain logistics | 36,172 | 644,375 | 83,944 |
| Cold-chain storage | 23,410 | 417,029 | 54,327 |
| Cold-chain wastage | 36,502 | 650,255 | 84,710 |
| **Total costs** | **1,044,355,971** | **1,057,173,404** | **1,061,543,962** |
| **Cost per delivery** | **10,444** | **10,572** | **10,615** |

Table S4. Incremental outcomes per woman for heat-stable carbetocin versus alternative prophylactic uterotonics by healthcare center type

A. Heat-Stable Carbetocin vs Oxytocin

| Healthcare center | Incremental costs (in $) | Deaths avoided | DALYs averted | All PPH events avoided | Severe PPH events avoided |
| --- | --- | --- | --- | --- | --- |
| All | -1.72 | 0.00005 | 0.0024 | 0.055 | 0.006 |
| Primary | -1.29 | 0.00007 | 0.0031 | 0.055 | 0.006 |
| Secondary | -1.77 | 0.00004 | 0.0023 | 0.055 | 0.006 |
| Tertiary | -2.42 | 0.00001 | 0.0015 | 0.054 | 0.006 |

B. Heat-Stable Carbetocin vs Misoprostol

| Healthcare center | Incremental costs (in $) | Deaths avoided | DALYs averted | All PPH events avoided | Severe PPH events avoided |
| --- | --- | --- | --- | --- | --- |
| All | -2.30 | 0.00010 | 0.0047 | 0.070 | 0.015 |
| Primary | -1.38 | 0.00015 | 0.0061 | 0.071 | 0.016 |
| Secondary | -2.55 | 0.00009 | 0.0045 | 0.070 | 0.015 |
| Tertiary | -3.66 | 0.00002 | 0.0025 | 0.070 | 0.015 |

1. Additional results for scenario and sensitivity analyses

Table S5. Scenario Analysis: Costs and health outcomes per woman for all births

|  | Total costs (in $) | Deaths | DALYs | All PPH events | Severe PPH events |
| --- | --- | --- | --- | --- | --- |
| ***Base case*** | | | | | |
| Heat-stable carbetocin | 139.90 | 0.00025 | 0.012 | 0.141 | 0.041 |
| Oxytocin | 141.62 | 0.00030 | 0.014 | 0.195 | 0.047 |
| Misoprostol | 142.20 | 0.00035 | 0.016 | 0.211 | 0.056 |
| ***Exclude cold-chain costs*** | | | | | |
| Heat-stable carbetocin | 139.89 | 0.00025 | 0.012 | 0.141 | 0.041 |
| Oxytocin | 141.39 | 0.00030 | 0.014 | 0.195 | 0.047 |
| Misoprostol | 142.17 | 0.00035 | 0.016 | 0.211 | 0.056 |
| ***Source for cold-chain costs: Telangana [1]*** | | | | | |
| Heat-stable carbetocin | 139.90 | 0.00025 | 0.012 | 0.141 | 0.041 |
| Oxytocin | 141.57 | 0.00030 | 0.014 | 0.195 | 0.047 |
| Misoprostol | 142.20 | 0.00035 | 0.016 | 0.211 | 0.056 |
| ***Source for cold-chain costs: Rajasthan [1]*** | | | | | |
| Heat-stable carbetocin | 139.90 | 0.00025 | 0.012 | 0.141 | 0.041 |
| Oxytocin | 141.66 | 0.00030 | 0.014 | 0.195 | 0.047 |
| Misoprostol | 142.21 | 0.00035 | 0.016 | 0.211 | 0.056 |
| ***Source for cold-chain costs: Diop and Vlasoff [2, 3]*** | | | | | |
| Heat-stable carbetocin | 139.95 | 0.00025 | 0.012 | 0.141 | 0.041 |
| Oxytocin | 142.44 | 0.00030 | 0.014 | 0.195 | 0.047 |
| Misoprostol | 142.31 | 0.00035 | 0.016 | 0.211 | 0.056 |
| ***Source for anemia:* WHO The World Bank Group [4] & 2019-2021 Indian National Family Health Survey – 5 [5]** | | | | | |
| Heat-stable carbetocin | 139.93 | 0.00025 | 0.012 | 0.140 | 0.041 |
| Oxytocin | 141.66 | 0.00030 | 0.014 | 0.195 | 0.047 |
| Misoprostol | 142.25 | 0.00035 | 0.016 | 0.210 | 0.056 |
| ***Source for efficacy vaginal births, additional uterotonics, blood transfusions: CHAMPION [6]*** | | | | | |
| Heat-stable carbetocin | 138.69 | 0.00036 | 0.014 | 0.143 | 0.031 |
| Oxytocin | 139.51 | 0.00039 | 0.015 | 0.168 | 0.033 |
|  | | | | | |
| ***No higher average dose of oxytocin to compensate for degradation of quality*** | | | | | |
| Heat-stable carbetocin | 139.90 | 0.00025 | 0.012 | 0.141 | 0.041 |
| Oxytocin | 141.44 | 0.00030 | 0.014 | 0.195 | 0.047 |
| Misoprostol | 142.20 | 0.00035 | 0.016 | 0.211 | 0.056 |
| ***Dosing for misoprostol: 400 mg*** | | | | | |
| Heat-stable carbetocin | 139.90 | 0.00025 | 0.012 | 0.141 | 0.041 |
| Oxytocin | 141.62 | 0.00030 | 0.014 | 0.195 | 0.047 |
| Misoprostol | 142.19 | 0.00035 | 0.016 | 0.211 | 0.056 |
| ***Costing method: apply 25% discount on dose price of oxytocin and misoprostol*** | | | | | |
| Heat-stable carbetocin | 139.88 | 0.00025 | 0.012 | 0.141 | 0.041 |
| Oxytocin | 141.46 | 0.00030 | 0.014 | 0.195 | 0.047 |
| Misoprostol | 142.15 | 0.00035 | 0.016 | 0.211 | 0.056 |
| ***Source for duration of hospital stay: Indian clinical expert opinion*** | | | | | |
| Heat-stable carbetocin | 144.61 | 0.00025 | 0.012 | 0.141 | 0.041 |
| Oxytocin | 151.97 | 0.00030 | 0.014 | 0.195 | 0.047 |
| Misoprostol | 155.82 | 0.00035 | 0.016 | 0.211 | 0.056 |
| ***Source of age distribution women: Seligman et al. (2006) [7]*** | | | | | |
| Heat-stable carbetocin | 139.90 | 0.00025 | 0.011 | 0.141 | 0.041 |
| Oxytocin | 141.62 | 0.00030 | 0.014 | 0.195 | 0.047 |
| Misoprostol | 142.20 | 0.00035 | 0.016 | 0.211 | 0.056 |
| **Key**: DALY, disability-adjusted life year; PHC, primary healthcare center; PPH, postpartum hemorrhage; SHC, secondary healthcare center; THC, tertiary healthcare center. | | | | | |

Fig S1. Tornado diagrams for heat-stable carbetocin vs oxytocin

A. DALYs Averted


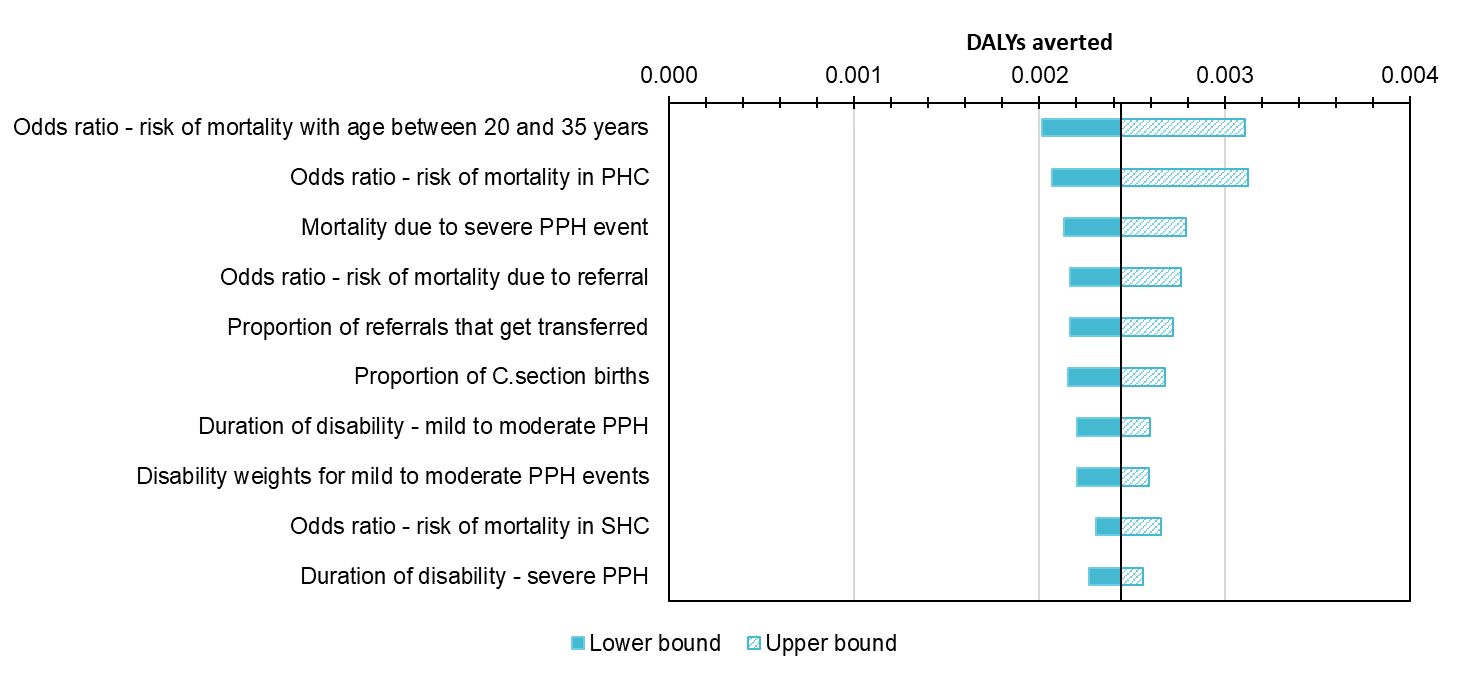


B. Incremental Costs (in US$)

**
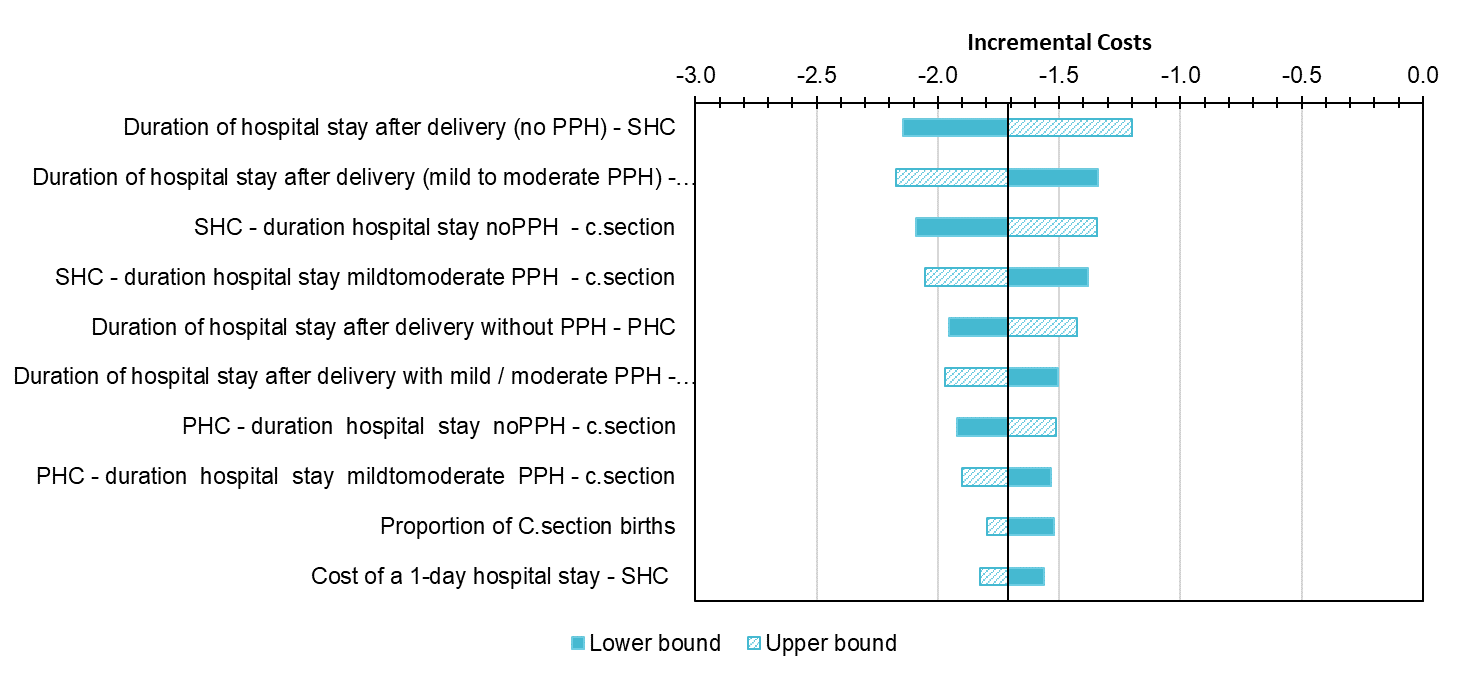
**

Fig S2. Tornado diagrams for heat-stable carbetocin vs misoprostol

A. DALYs Averted


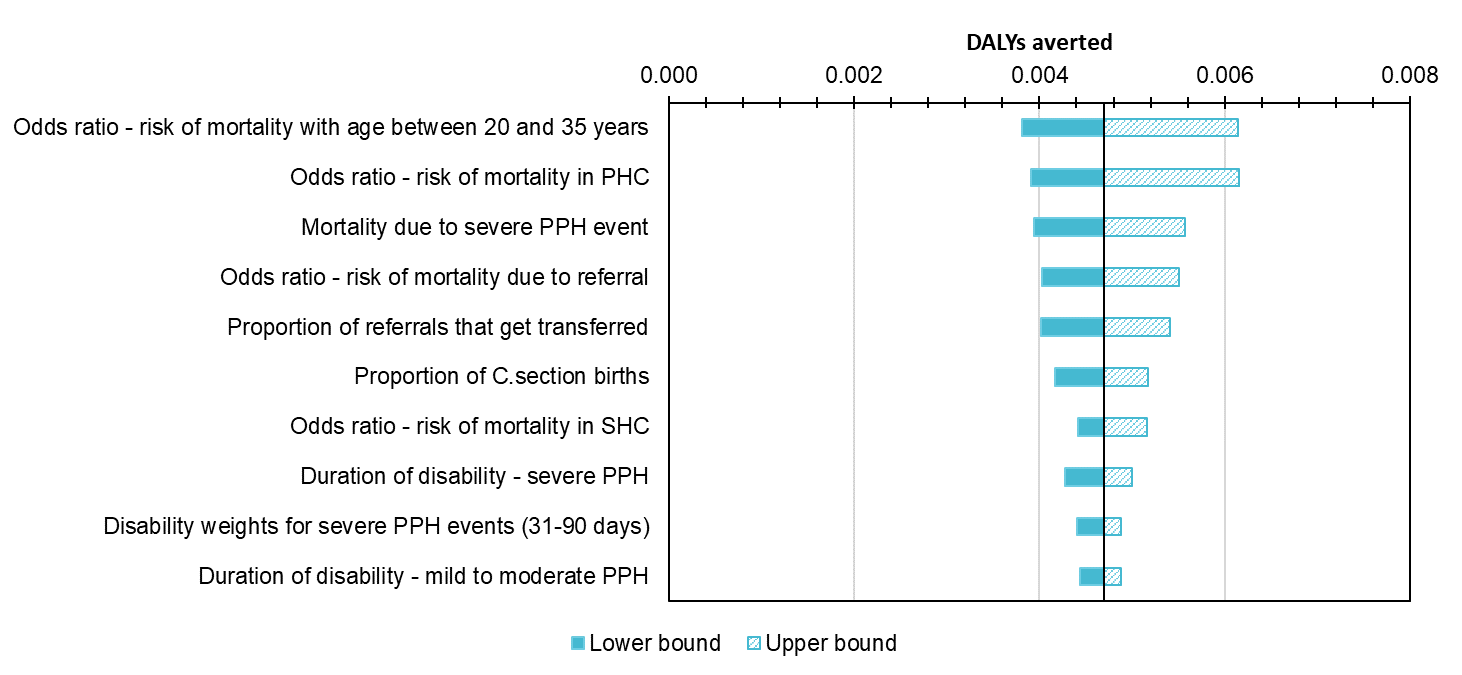


B. Incremental Costs (in US$)


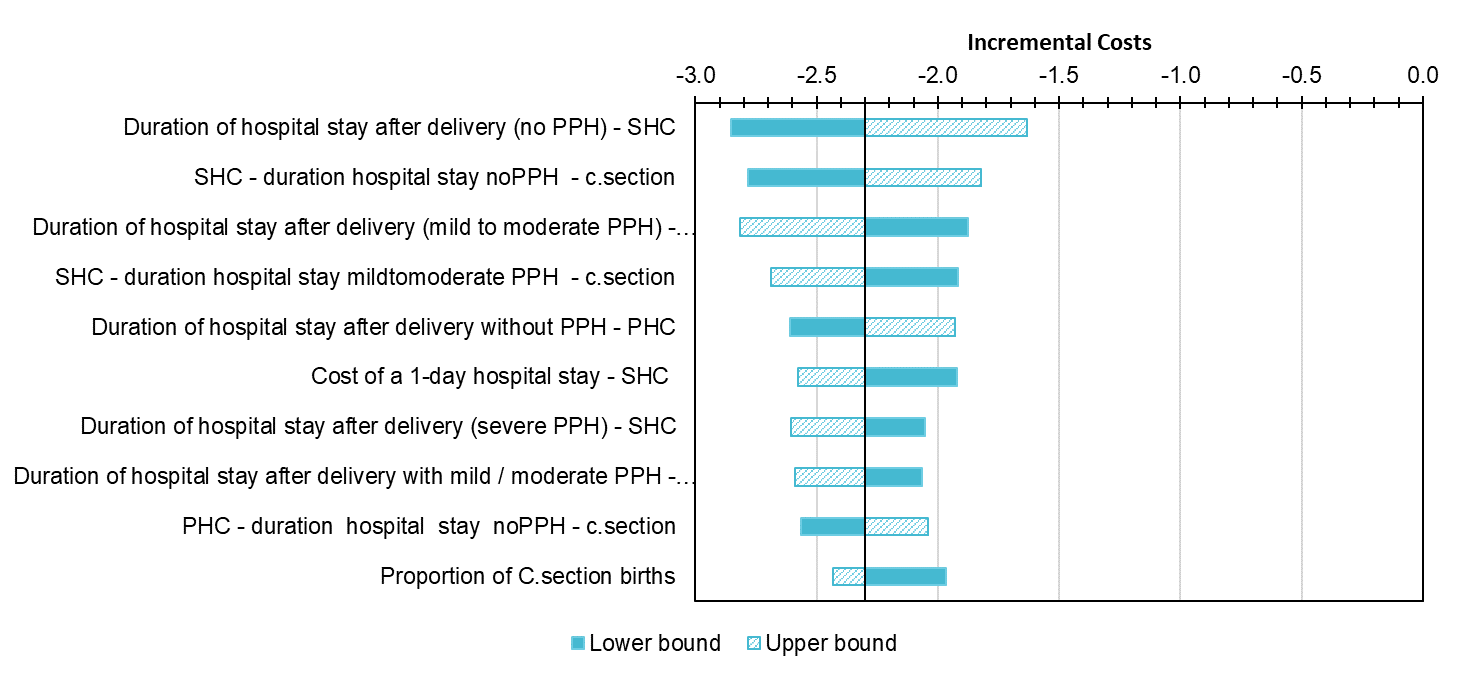


References

1. ACCESS Health International. 2018. Estimation of cold chain of Oxytocin in Rajasthan and Telangana. Data on file with Concept Foundation, Geneva Switzerland.
2. Diop A, Daff B, Sow M, et al. Oxytocin via Uniject (a prefilled single-use injection) versus oral misoprostol for prevention of postpartum haemorrhage at the community level: a cluster-randomised controlled trial. Lancet Glob Health. 2016; 4:e37-44.
3. Vlasoff M, Diallo A, Philbin J, Kost K, Bankole A. Cost-effectiveness of two interventions for the prevention of postpartum hemorrhage in Senegal. Int J Gynecol Obstet. 2016; 133:307–11.
4. The World Bank Group. Prevalence of anemia among pregnant women (%). 2019 April 3. Available from: <https://data.worldbank.org/indicator/SH.PRG.ANEM?locations=IN>.
5. International Institute for Population Sciences (IIPS), Mumbai. National Family Health Survey – 5 (2019–2021), Ministry of Health & Family Welfare, Government of India. 2021 November 4. Available from: <https://ruralindiaonline.org/en/library/resource/national-family-health-survey-nfhs-5-2019-21-compendium-of-fact-sheets-key-indicators---india-and-14-statesuts-phase-ii/>.
6. Salomon JA, Haagsma JA, Davis A, et al. Disability weights for the global burden of disease 2013 study. Lancet Global Health. 2015; 3(11):e712–23.
7. Seligman B, Xingzhu L. Economic assessment of interventions for reducing postpartum hemorrhage in developing countries. 2017 February 14. Available from: <http://abtassociates.com/reports/2006/economic-assessment-of-interventions-for-reducing.aspx>.
